# Hardware Security of Fog End-Devices for the Internet of Things

**DOI:** 10.3390/s20205729

**Published:** 2020-10-09

**Authors:** Ismail Butun, Alparslan Sari, Patrik Österberg

**Affiliations:** 1 Department of Computer Engineering, Chalmers University of Technology, SE-412 96 Göteborg, Sweden; 2 Department of Computer Engineering, Konya Food and Agriculture University, 42080 Konya, Turkey; 3 Department of Electrical and Computer Engineering, University of Delaware, Newark, DE 19716, USA; 4 Department of Information Systems and Technology, Mid Sweden University, 851 70 Sundsvall, Sweden; patrik.osterberg@miun.se

**Keywords:** cloud, edge, fog, IoT, IIoT, privacy, protection, HSM, PUF, TRM, SoC

## Abstract

The proliferation of the Internet of Things (IoT) caused new application needs to emerge as rapid response ability is missing in the current IoT end-devices. Therefore, *Fog Computing* has been proposed to be an edge component for the IoT networks as a remedy to this problem. In recent times, cyber-attacks are on the rise, especially towards infrastructure-less networks, such as IoT. Many botnet attack variants (Mirai, Torii, etc.) have shown that the tiny microdevices at the lower spectrum of the network are becoming a valued participant of a botnet, for further executing more sophisticated attacks against infrastructural networks. As such, the fog devices also need to be secured against cyber-attacks, not only software-wise, but also from hardware alterations and manipulations. Hence, this article first highlights the importance and benefits of fog computing for IoT networks, then investigates the means of providing hardware security to these devices with an enriched literature review, including but not limited to Hardware Security Module, Physically Unclonable Function, System on a Chip, and Tamper Resistant Memory.

## 1. Introduction

The Internet of Things (IoT) is having its boom phase now, as the Internet had two decades ago. The IoT market is growing and expected to increase from more than 15 billion devices in 2016 to more than 75 billion by 2025 [1]. Following this trend, the number of deployed IoT devices has already passed the total population of Earth. Furthermore, in the last decade, the proliferation of mobile computing has expanded exponentially. It is expected to continue its pace this way to result in each person on Earth having an average of six connected devices [2]. In order to keep this rapid growth and the huge consumer market it possesses, IoT needs a rigid technological foundation supported by the scientific community. Fog computing is a very strong candidate to provide this foundation (in parts or totally) for IoT, by providing several advantages, in terms of computational, architectural, and networking point of view [3].

Emerging from recent trends and needs, cloud computing and IoT will serve as complementary technologies of the Internet in the near future, by forming the concept called Cloud of Things (CoT). CoT will be leveraged as Things as a Service (TaaS) for cloud based IoT applications, for offloading high energy consuming tasks and operations to the cloud. TaaS will support innovative scenarios and use cases, breath-taking services along with ubiquitous and value added applications to enable CoT to be accessible by the users. In the meantime, fog computing and all virtual/real services associated with it can be thought of an intermediate layer to rapidly process the data at the edge of the network, serving the fast response need of the agile applications [2,4].

The fog layer can also leveraged as the *security layer* to provide necessary privacy and security functions to protect the data before it is offloaded to the cloud through insecure and vulnerable channel [2].

### 1.1. Why Security Is Crucial for IoT and Fog?

As being a centralized resource out of users’ reach and control, the cloud computing environment represents every possible opportunity to violate user privacy. Undoubtedly, privacy is becoming a desired luxury today, a situation that will be exacerbated with the proliferation of the IoT devices everywhere surrounding us [5]. We have started to observe more IoT security related news than ever. For instance, Mirai and its’ variant botnet attacks have shown that IoT botnets can be very effective with large-scale deployments to execute Distributed Denial of Service (DDoS) attacks. Recently, Japan announced that 200 million deployed IoT devices were going to be investigated by white-hat hackers. These detectives will try to log into devices which are accessible through the Internet by using publicly known default credentials. In order to foresee and identify unexpected cyber-security threats towards IoT, this security trial is scheduled to happen in March 2020, just before the Summer Olympics in Tokyo [6].

As discussed in this text, fog computing is also becoming an integrated part of the IoT networks. Hence, the privacy issues are not solved with it but maybe multiplied—in terms of complexity—the ownership of the data that is being produced, transferred, and processed. Therefore, this article investigates the remedy that is needed to address the privacy and security concerns of the users in fog computing supported IoT networks.

### 1.2. Which One Should Be Preferred for IoT: Cloud, Mobile-Edge, or Fog?

In recent years, due to the usage of IoT and other sensors, the data generated by end-devices increased massively. The question is where, when, and how should these data be analyzed.

In cloud-centric design, the cloud server operates as a central server. IoT devices generate the data and send them to the cloud for storage and analysis. Large-scale IoT deployments create situations which cloud computing could not handle efficiently and effectively.

However, in fog computing, the data are to be analyzed on the edge stations and just necessary results (summaries) are being sent to the cloud server for further analysis and storage. For instance, applications which require low latency while processing the data on the edge of the network might benefit from this technology. The data analysis could be done on site by running the software at local stations. The cloud would still be used as storing the analysis result for historical and audit purposes. The data aggregation will reduce the bandwidth and also bandwidth related cost.

The fog computing concept was introduced by CISCO [7] and was a vision that enabled IoT devices to run on the edge of the network. According to Bonomi et al. [8], fog computing is not an alternative for cloud computing; instead, fog extends and complements the cloud computing with the concept of smart devices which can work on the edge of the network.

In IoT, with different types of data generated by various heterogeneous nodes, inseparability issues arise as an important problem. Fog computing can provide remedies to this problem by handling trans-coding related specific tasks at the edge of the network [2].

**Figure 1:**
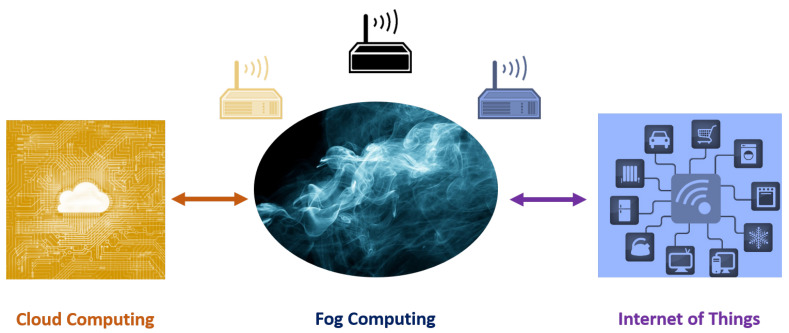
Fog computing as a gateway in between cloud computing and IoT.

As shown in Figure 1, fog computing can be thought of a gateway between cloud computing and IoT, for the sake of enhancing the Quality of Service (QoS) in some specific applications such as Industrial Internet of Things (IIoT), where rapid and agile response is of prime importance. Fog computing also projected to provide remedies to long known problems and challenges of the cloud, namely: data aggregation and processing from heterogeneous devices along with interoperability issues of those devices; data protection and security of the sensitive user data; context-aware and location-aware service provisioning especially for the location-based services (LBS).

The use of the cloud paradigm is where data need to be collected first and transmitted to a central location to store and analyze later due to hardware constraints on edge. The Fog/Mobile-Edge paradigm is preferred when data are collected on the edge and needs to be processed immediately to eliminate latency or preserve availability. For instance, in avionics concepts, the cloud paradigm is preferred with space crafts and orbital satellites since, due to hardware limitations, data are collected on the edge and transmitted to earth for storage and further data analysis. In the case of commercial airlines, fog/mobile-edge would be advantageous since various analog sensors were deployed to the plane and need to be digitized to feed mission computer and inform pilots for decision. Therefore, data need to be consumed on edge rather than transmitting to a central location to get decisions.

### 1.3. Why Hardware Assisted Security for Fog?

The proliferation of IoT devices such as sensors has resulted in high data bandwidth demand from the IoT network to the cloud due to the vast amount of data being produced and transferred. Fog computing is proposed to provide a remedy to this challenging and growing problem: Instead of transferring all of the IoT data to the cloud, fog computing will process the data at the edge. Fog computing brings most of the advantages and benefits that cloud computing offers down to the edge of the network that will be available to IoT end-devices and users. However, this integration will bring many new challenges for the researchers, especially while building cyber-security related solutions. Therefore, this integration needs to be supported from the cyber-security point of view. One way of doing is leveraging commodity hardware security platforms such as Hardware Security Module (HSM) and Physically Unclonable Functions (PUF). This article investigates efficient and seamless implementations of this kind.

### 1.4. Demystifying Fog Computing

In an earlier publication [9], we have mentioned the security implications of fog computing for IoT networks. Here, in this manuscript, we focus on the fog computing devices and, to enhance the security of those, we stress more about the hardware platforms that can be leveraged.

As shown in Figure 1, from a conceptual point of view, fog computing might be expected to serve as an intermediate level of service for flawlessly handshaking the protocols of cloud computing and IoT. Sometimes, this service is referred to as Fog as a Service (FaaS) in the literature. FaaS will bring many benefits to IoT and its users: (1) Servers of the cloud computing are super fast when compared to the IoT end-devices. Fog Computing Gateways (FCGs) would provide an interface between the two far sets of those devices. (2) This intermediate layer of fog computing would allow necessary fixes (such as patch management, etc.) to be done easier and remotely. Instead of making the configurations on IoT end-devices by plugging-in physically, software updates can be pushed on to the fog gateways which then deliver the patches to intended end-devices. (3) Fog computing will bring all the advantages of edge-computing to the IoT, such as the agility, scalability, decentralization, etc. (4) Fog will expand clouds to provide additional assets to the underlying nodes and networks by taking advantage of virtualization concept by creating virtual sensors and networks to be used by other various services. (5) Finally, fog enables and creates an environment for proliferation of distributed IoT applications.

### 1.5. Content and Scope

In this article, our aim is to find, identify, and discuss available COTS and/or conceptual hardware solutions for securing low-end devices of fog-based IoT networks. To facilitate this, we first presented the fog computing concept and the advantages it would bring to IoT networks. Cyber threats against IoT networks are presented in Section 2.4, and the hardware attacks are in Section 4.1. Implications of using fog-computing based IoT networks are presented. This is followed by the remedies that would be offered by hardware assisted security techniques such as TRM, PUF, HSM, etc. This is followed by practical real-life scenarios in which fog based IoT networks can be supported by hardware-based cyber-security solutions.

### 1.6. Organization

The rest of the manuscript is provided as follows: Section 2 presents the concepts of fog, mobile-edge and cloud computing in a comparative way. Section 3 discusses the implications of using fog computing for IoT in terms of systems integration, cost, QoS, consumer needs, and security. Hardware assisted security solutions for fog computing devices are discussed in Section 4. Some practical application scenarios of fog computing supported IoT network are presented in Section 5. Finally, Section 6 concludes the paper along with future remarks.

## 2. Fog vs. Cloud/Mobile-Edge Computing

Major distinctions between cloud, fog and mobile-edge computing are provided in Munir et al.’s work [10], Stallings’ work [11], and Luan et al.’s work [12]. We extended all these as discussed below and tabulated them as shown in Table 1.

### 2.1. Fog vs. Cloud Computing

Fog and cloud computing are different paradigms; they are not rivals but complimentary to each other to build up a stronger and agile network. The basic trade-offs between fog and cloud are provided in Aazam et al. [2] as follows:

- communication efficiency
- total power consumption for a service
- round-trip response time for a query or task.

Here, we extend this list as follows:

- Place of data processing: Fog computing implements the idea of bringing the functionalities of cloud computing to the data source. This is an analogy to meteorology, as fog is simply a type of cloud that is closer to earth. Henceforth, fog computing extends the services of the cloud computing downward towards the edge of the network.
- Proximity to the users: FCGs are very close to the IoT users and end-devices, whereas cloud computing is executed over servers located at far side from the IoT users.
- Network delay: The cloud computing servers are most generally at least several hops away from the IoT users and end-devices. Therefore, in some cases, a round trip of communication (bi-directional) may last in the neighborhood of seconds. On the contrary, owing to the off loaded server architecture, fog computing may receive and respond the queries from the IoT network in milliseconds. Henceforth, they are very promising for agile applications such as IIoT and CPS.
- Location-Based Services (LBS): One of the major benefits of fog computing over the cloud is that the support for location awareness which might be very useful for the applications that are employing LBS.
- Mobility Support: Mobility is fully supported in fog computing by leveraging virtual machine (VM) technologies. However, for cloud computing, mobility of the users is supported in a very limited way.

### 2.2. Fog vs. Mobile-Edge Computing

Due to a misconception, *fog computing* and *mobile-edge computing* are sometimes being used interchangeably in the literature. However, they differ in the following ways:

- Decentralization: Fog computing provides more decentralized and distributed architecture when compared to mobile-edge computing in which generally cellular base stations are the main point of centralization.
- Supplier diversity: In mobile-edge computing, the hardware/software components are supplier specific and there is no standardization in the market. For fog computing, this is not acceptable. System cost, quality, innovation, market adoption and proliferation of fog computing are all dependent on standardization.
- Diverse Radio Access: Most of the mobile-edge computing applications are for mobile and/or cellular networks, whereas fog computing will include WiFi, LPWAN, and WiMax additional to the cellular network.

Connectivity of large number of IoT devices and various latency requirements of them will be seamlessly supported by the massive Machine Type Communication (MTC). Non-Orthogonal Multiple Access (NOMA) technology, and, more specifically, Mobile Edge Computing (MEC) has the advantages of improving network capacity, reducing MTC devices’ latency, and enhancing Quality of Service (QoS) [14].

### 2.3. Advantages of Using Fog Computing for IoT

Table 1 presented fog, mobile-edge, and cloud computing concepts in a comparative way. As can be seen, fog computing presents a more agile and rapid response when compared to mobile-edge and cloud computing, thereby represents a strong candidate as a technological solution for future IoT and IIoT based implementations.

As mentioned earlier, fog computing can be considered as an extension of the cloud computing towards the edge of the IoT network with an increased agility. Thereby, fog computing offers the following advantages when used for IoT (and IIoT) networks:

- Cost efficiency: The data will be processed on edge rather than cloud which will eventually decrease transportation of huge amount of data to the cloud along with the associated cost.
- Support for interoperability: Fog devices can help with trans-coding related tasks to relieve the problem of the interoperability of the heterogeneous IoT end-devices [2].
- Reduced delay: The cloud computing is not suitable to serve for time-critical applications such as for IIoT, as overall end-to-end delay is in the neighborhood of 100 ms (which is critically high, especially for factory automation that require isochronous response in the low milliseconds [15]). As being located at the edge of the network, fog computing is a strong candidate to provide faster communication and thereby reduced delay for the communication packets.
- Agile response: Real-time applications, such as IIoT, may benefit from the fog computing concept to gain agility during analysis and decision-making phases of their overall process automation cycle.
- Increased security: With fog computing, service providers can easily filter out sensitive personally identifiable information (PII) and process them locally. Instead of sending all the information, only the non-sensitive information is sent to the cloud for further processing [16].

### 2.4. Cyber-Attacks and Ways of Protecting the Networks from Them

In the literature, many attacks have been identified in IoT networks such as Mirai and Torii botnet attacks [9], and various attacks against industrial networks (IIoT, etc.) such as Stealthy attacks [17]. Henceforth, in order to have solid and operable systems and to cope with cyber-attacks, cyber-security measures need to be taken. As mentioned in Butun et al. [18], cyber-security of any computer system consists of three layers: *Prevention*, *Detection*, and *Mitigation*.

On the intrusion detection side, anomaly detection is a very commonly used technique. Anomaly detection based on device or event logs and process model (cyber threats and faults) is another important aspect to prevent possible attack vectors crafted for the targeted systems. For instance, Audi investigated the intruder detection and monitoring approaches to secure the Industrial Control Systems based on anomaly detection [19]. In a similar work on industrial networks, Myers proposed a method to detect cyber attacks on industrial control systems using process mining [20]. However, MITM, hardware hijacking, etc. attacks are omitted in this study. In another work, Aydogan et al. [21] investigated intrusion detection systems under *Hello flood* and *Version number* attacks for RPL-based IIoT.

In Greenberg et al. [22], the following statement is really disconcerting: “A new proof-of-concept hardware implant shows how easy it may be to hide malicious chips inside IT equipment.” This is a very good proof showing that, not only the software systems, but also commodity hardware platforms are vulnerable to attacks. These are sometimes also referred to as ‘hardware-hacks’ and remind system developers to include hardware level authenticity validations right after factory production line, followed by before and after installment validation procedures.

This work focuses on the *Prevention* mechanisms to thwart attackers in the first place before any attack can happen. On the cyber-prevention side, trusted hardware components might be utilized: Smart Environment Monitoring (SEN) systems are on the rise and many various applications are being developed to monitor sensed environment data via IoT [23]. Hence, the reliability of these data is important and supports our standing point here, as trusted hardware platforms can fulfill the necessity of ‘reliable data’.

### 2.5. Why Is Security in Fog Computing Needed?

**Figure 2:**
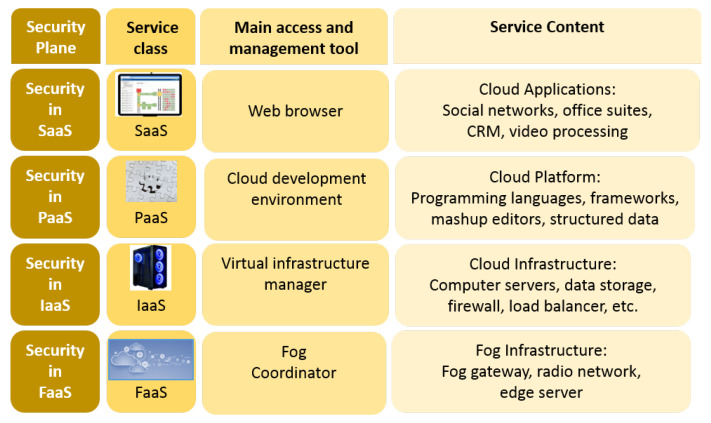
Representation of “Fog as a Service” model.

Fog computing is actually a tool for Cloud-Based Services (CBS) that can be imagined as an interface between the real end-devices of the IoT and the rest of the CBS. As discussed in Butun et al. [24], CBS offers three major service components, namely Infrastructure as a Service (IaaS), Platform as a Service (PaaS), and Software as a Service (SaaS). We are projecting that fog computing paradigm will extend this presentation by including Fog as a Service (FaaS) as the 4th component to the service model as shown in Figure 2.

The *Security Plane* for CBS proposed by Butun et al. [24] was devised to be used at the front-end IoT devices and to provide an interface to the cloud. After the proposal of fog computing, this *Security Plane* solution is more diverse and layered compared to an earlier version (see Figure 2). Therefore, we think of fog computing to provide extra services such as security to the edge of the cloud for the CBS. For example, the usage of fog computing would bring benefits to the Intrusion Detection Systems (IDS) that are devised for IoT. Hence, early detection is important to stop the ill effects of intrusions, fog computing would bring early detection opportunities to IDS algorithms working on IoT. It is worthwhile to mention that the security plane of Figure 2 will be incomplete, unless all necessary security and privacy functionalities are implemented accordingly.

## 3. Implications of Fog Computing Usage in IoT

According to the scientific projections, fog computing is expected to be one of the main backbone pillars of the IoT in the near future transforming the cloud computing based IoTs to a more distributed architecture [1,16,25]. Inevitably, there will be some implications of this transformation as follows:

### 3.1. Related to Systems and/or Subsystems Integration

Systems and/or subsystems integration is an important task for the systems engineers as their duty is to provide all components of a newly built system to work seamlessly. As such, the introduced system (the IoT security components, etc. in our case) should be both compatible with cloud computing servers from the high-end, and also with the IoT end-devices (sensors and actuators) from the low-end. It should not create an extra burden for network managers in setting up and configuring the fog computing devices.

### 3.2. Related to Telecommunications

**Figure 3:**
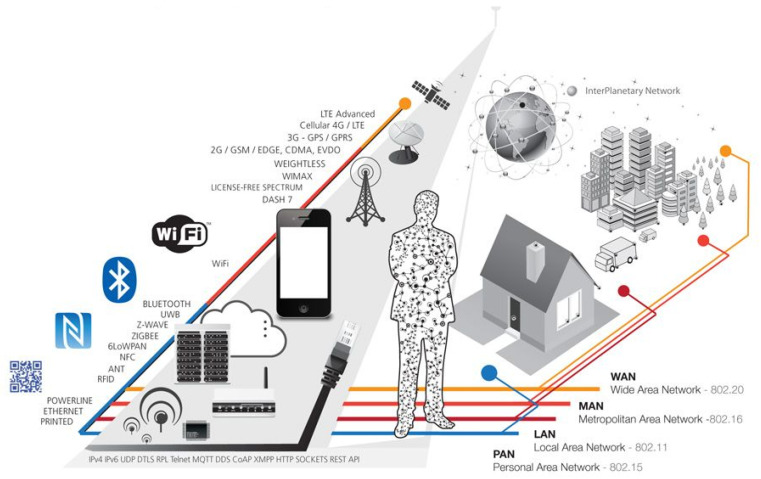
IoT connectivity diagram [26].

Table 2 shows the IoT related telecommunication (RF) technologies in a comprehensive manner. The selection of the RF technologies can be based on the intended functionality and on the hardware requirements such as bandwidth, cost, energy efficiency, latency, and network type, etc. As discussed in Figure 3, these RF technologies establish the foundation for the IoT networking technologies. The details of these communication and networking technologies are beyond the scope of this article (Interested audience may refer to [13] for further reading).

Besides the great benefits of having a diverse spectrum of RF technologies, orchestration of these technologies and making them work in harmony require tremendous effort, especially from the cyber-security point of view. Hence, one of the goals of this paper is shedding light on the cyber-security related dark spots of the fog-computing associated technologies.

### 3.3. Related to Cost

Recently, United Parcel Service (UPS) deployed IoT sensors to collect data for data analysis and optimization to reduce the cost and to improve the efficiency in their postal distribution system [27]. According to Jalali et al. [28], fog computing may help to reduce the energy cost in cloud computing systems. Therefore, we foresee that fog computing will help cloud computing-supported IoT systems to decrease their overall system costs. As the data will be processed on edge rather than the cloud, fog would reduce cost overheads of transmitting data to the cloud. This will have two benefits: (1) Bandwidth usage of the transmission will decrease drastically. (2) The size of the data storage at the cloud (along with the processing) will decrease.

### 3.4. Related to QoS

QoS is an important service criteria that eventually affects the satisfaction of the users while using the provided service. For IoT, the temporary unavailability of the sensors or actuators within IoT applications will directly impact the physical world and hence drastically decrease the QoS for the network users [5]. Many existing wireless applications have diverse and mandated quality of service (QoS) requirements, which complicates the burden on the integration of IoT and cloud. In the meantime, emerging fog based middle-ware solutions might be useful and handy in that sense to offer handling of urgent tasks at the edge of the network and offloading data from energy scarce nodes [2].

As mentioned in Lai et al. [29] and Yi et al. [30], fog computing can drastically improve the QoS of IoT networks by decreasing the ‘packet latency’ and ‘network congestion’ while increasing the ‘failure detection’ and ‘loss recovery’.

### 3.5. Related to Security

The real-life environment practically differs from ideal settings. Extra features and components sometimes create single point of failures or put toil (e.g., processing, memory storage, power consumption, etc.) on the existing system. In well designed and planned implementations, single point of failures and the toil on the system can be eliminated to improve overall system performance as desired. This is valid for both hardware, software and security features associated with the inclusion of an extra component.

Several key security considerations for cloud-based IoT networks were addressed by Singh et al. [31], which focused on security-related to IoT and cloud integration such as auditing, software issues, policy, protocols, trust, etc. However, they did not touch any of the security problems from the hardware perspective.

Security implications of using fog computing for IoT systems are described below. There are 6 features that we considered in case of a capture of the FCG device:

1. Access Control: FCG connects IoT/IIoT networks and cloud with a bidirectional communication channel. The data are collected and streamed from IoT devices to cloud, and decision and command messages sent from cloud to IoT networks. An FCG device can manage all connect IoT devices in an efficient manner. However, FCG devices can not directly access to databases and other computational resources without a designated cloud service.
2. Authentication: An FCG device may have some implications based on the selected authentication algorithm. If the authentication is designed to operate by just FCG devices, it increases the risk of getting all the IoT network being compromised once the FCG device is hacked. Usually two-factor authentication and multi-layered authentication (one at FCG, one at the cloud, etc.) mitigates the risk of an FCG is being compromised. In case of an incident, only a subset of connected IoT devices would be effected.
3. Availability: Cloud resources are more resistant to single point of failures. Data are replicated on multiple nodes on cloud and a fail over event can be achieved seamlessly. However, IoT devices are more susceptible to disruptions such as if any communications are blocked for IoT resources, it may cause a significant impact on availability based on the critical location of the FCG.
4. Confidentiality: Compared to cloud, the data confidentiality on FCG has a moderate impact. The devices connected to FCG has impacted directly, but the rest of the IoT devices and cloud should not be impacted by it.
5. Integrity: Based on the selected communications scheme, the minor effect of a FCG capture on the integrity of the messages is expected if end-to-end encryption is not employed, to no effect if encryption is employed.
6. Privacy: Any data leak and privacy violation of users in IoT networks via an FCG device is a serious problem not limited to reputation, financial loss, or other implications to organizations. Any user which is using an IoT device or stored data on IoT device would get exposed via a hacked FCG device. However, the private data on cloud would not get impacted with this.

All the communication between the cloud and IoT is flowing through FCGs. Therefore, an adequate amount of computers need to be installed to prevent a single point of failures and handle self-heals and fail-over events seamlessly. Since all the data flow would be blocked in case of any damage or a physical attack, we suggest several FCGs to be installed in the fog computing supported IoT network architectures with the capability of self-healing and seamless fail-over.

A compromised FCG affects both the IoT network and cloud layer at low to critical levels depending on the situation and security mechanisms implemented which are explained above. The security of FCG is important and should not be negligent to leave unprotected. As the next section summarizes (see Section 4), hardware assisted security should be leveraged to add on FCG as an additional defence layer on top of software oriented solutions.

Firewall and/or IDS components should be integrated into the fog layer to implement a preemptive security mechanism. A well configured firewall can prevent most of the attacks and an IDS can detect anomalies in the network to detect intrusion attempts. IDS can also be utilized with different technologies like Markov model, virtual honeypot device, etc. [32]. Moreover, the anomaly detection concept using various components such as autoencoders and neural networks to apply deep learning algorithms to identify new trends on edge could be another defense layer variation for the IoT network [33]. Therefore, leveraging security service prevents a propagation of attacks further to the cloud.

The practice of just sending the minimal amount of data to the cloud and keeping data on the edge would mitigate data privacy and security related issues.

## 4. Hardware Assisted Security of Fog Computing Devices

Arias et al. [34] introduced a case study that concluded that a non-secure hardware platform would lead to a non-secure software stack, hence highlighting the importance of hardware security. Kaur et al. [35] also mentioned that “Hardware Security” is not well studied in the literature when compared to software-related cyber-security problems and solutions. Recently, there are emerging studies on hardware assisted security on IoT related devices such as: building a unified identity verification framework based on PUFs [36], FPGA hardware security for data centers [37], and re-configurable hardware-based isolation and protection mechanism (IPM) for IoT devices using cloud [38]. All these support our effort in introducing hardware security measures for FCGs. This section summarizes all possible hardware security measures that can be considered for the FCGs, while deciding security provisioning for the overall IoT network:

### 4.1. Threat Model and Security Risk Analysis

**Figure 4:**
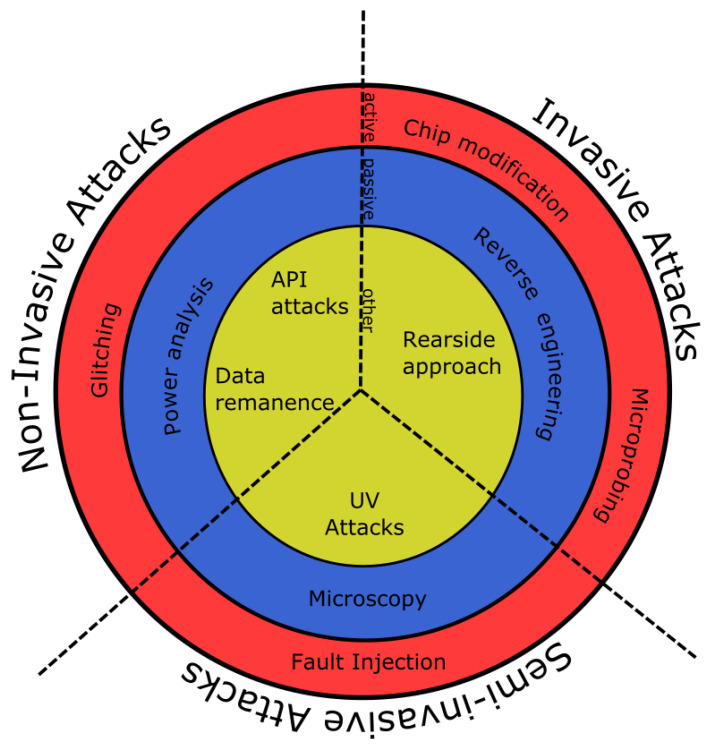
A subset of hardware related attack vectors and their relations [39].

As discussed in Skorobogatov et al. [39], cyber-security attacks against hardware platforms can be investigated under three groups: invasive, non-invasive, and semi-invasive. Among these, the most severe one is the invasive one as it directly intervenes with the operational structure of the circuits on the hardware. Figure 4 shows further details and classifications of these attacks.

The reverse engineering method is used to uncover the hardware’s physical properties and functionalities to reproduce the technology or exploit possible vulnerabilities. Power analysis and eavesdropping techniques can be correlated with RE to learn more about target hardware’s properties. These attacks are mostly used in industrial espionage to steal competitors technology. Microprobing and chip modifications are also used for monitoring and manipulating the hardware unit while interfering with the integrated circuit directly. Software attacks target code level vulnerabilities or weaknesses including business logic and code implementations, etc. Fault injection or generation can be used to violate the CIA (confidentiality, integrity, availability) principle such as making a device to malfunction to gain access, stop functioning, or perform other critical tasks. Glitch attacks like clock or power glitches can be used create a possible vulnerability window to perform a specially crafted attack. Data remanence is the concept of deciphering information bits in physical devices like memory (SRAM, DRAM, EPROM, etc.) and storage units to retrieve security keys or other critical information bits.

The focus of hardware security should be at least addressing C (Confidentiality) and I (Integrity). Hidden information about the device or data should not be exposed and tampering should be prevented. In the industry, secret information (property, data) is commonly attached to a physical object and hardware must protect it. As an example, smart cards are used for multiple tasks such as accessing a facility or keeping monetary information, etc. and a simple cloning attack may have an immediate impact [9]. In the case of cloning being successful for bank cards, the financial loss would be devastating.

Generally, the following techniques can be considered to prevent or slow down hardware related attacks as the first layer of the defense; obfuscation: data (deletion or nulling out, encryption, masking, substitution, shuffling and other complex techniques, etc.), layout (by using 3D stacking and complex architectural design to protect chip integrity) and metal mesh (to prevent probing attacks). Finally, using physical shields to protect hardware from outside noises to prevent fault injection attacks. Table 3 shows security risk analysis for hardware cyber-threats vs. common security properties for fog-computing based IoT end-devices including attack vector classification [40]. For instance, a security risk analysis of rogue end-device (i.e., a captured end-device by the adversaries) with respect to common security properties is as follows: C has moderate, I has moderate, A has moderate, and Auth* has significant impact. The associated attack vector is In or S.

It has been observed in this subsection that there are several obstacles to be tackled in order to accommodate cyber-security for fog-based IoT devices via hardware-assisted solutions due to inherent problems of the IoT and the end-devices themselves: Physical access vs. remote access (hardware solutions would require physical access to the real device which sometimes might bring a real burden on the field teams/technicians), flexibility (hardware systems are not very much flexible as software systems; although some hardware platforms are re-configurable such as FPGAs, they are very costly in that form), scalability (due to hardware limitations, cost, and other production constraints’ hardware solutions are hard to scale), agility (time required to install hardware fixes might be significant).

### 4.2. Resistance against Reverse Engineering (RE)

‘Tamper resistance’ means that taking measures to make Reverse Engineering (RE) harder for attackers or to prevent them from modifying a product against the producer’s will. This can be achieved in three ways:

#### 4.2.1. By-Software

Software solution for tamper resistance would contain ways of shuffling the executable code on the memory so that it would not reveal specific information regarding any confidential and meaningful material to the attackers. One commonly used method for doing this is code obfuscation [41]. Historically, white box implementation [42], dynamic program monitoring [43], self-hashing [44], and check-summing [45] are provided as methods for tamper resistance. Finally, blockchain technology is a viable option to satisfy data integrity in the IoT network using consensus [46] strategy between nodes. As a proof of concept, a scheme of blockchain integrated PUFs in IoT is proposed in [47], using a unique consensus algorithm called “Proof of PUF-Enabled Authentication”.

#### 4.2.2. By-Hardware

The discussion on attack vectors against cryptographic hardware (smart cards and micro-controllers) and the need of tamper proof systems dates back to the 1990s [48]. Chip-based protection is also designed to protect intellectual property. Game console, phone, or other device hacks to run illegal copies of the software the designated device is another motivation for tamper proof systems [49]. A subset of known attack vectors to hardware are differential fault analysis, chip rewriting, memory remanence, and protocol failures [50]. Logic locking of Boolean circuits can be used as a preventative method [51].

Tamper Resistant Memory (TRM) chips may be designed to delete their sensitive data including cryptographic keys, if they can detect an intrusion towards their security encapsulation. The working mechanism behind this is continuously updating the memory cells to prevent static data imprint on them. TRM is mostly used to store sensitive information like private keys, electronic payment information, etc.

Another approach would be implementation of a hardware-based System on a Chip (SoC) concept. For instance, Da Silva et al. [52] proposed an SoC to protect low-end embedded processors from control flow attacks, especially from the Code Reuse Attacks (CRAs). The proposed concept provides an end-to-end protection combination of detection, response, recovery, and tamper evident techniques against control flow violation (caused by CRAs), especially in the presence of interrupts, real-time operating systems, and exceptional functions.

#### 4.2.3. By-Design

IoT frameworks are dependent on underlying hardware and smart electronics as gateway devices such as sensors and micro-controllers. These may be subjected to malicious Hardware Trojan (HT) inserted by the untrusted chip manufacturers. An HT is strategically inserted into normal looking hardware by using RE skills and acts as time-bomb: It is suddenly activated during a normal operation mode of the hardware, and may cause abnormal and unintended operation. Defense against this kind of attack can be one of the popular techniques such as ‘functional and structural obfuscation’ which is executed by the authentic device manufacturers before the fabrication phase of the chips. HT also can inject faults to create DoS attacks to starve the target network [53]. HT detection is quite challenging due to involvement of many third party intellectual property related to manufacturing business [54].

### 4.3. Physically Unclonable Functions (PUF)

PUF is a physical object which provides a digital fingerprint for hardware like microprocessors based on various inputs and challenges. Silicon circuits manufacturing results with unique characteristics of the products which can not be physically cloned [55]. Each PUF circuit should have a unique Challenge Response Pair (CRP) which can be used for identification and authentication purposes [56,57]. Fabrication and architectural details of weak (has small number of CRPs) [58,59] and strong (has lots of CRPs) [59] PUFs are also important. Strong PUFs are resistant to brute force attacks. The main characteristics of PUFs are reliability (it should always provide same CRPs), unpredictability (PUF CRPs should not be predicted based on other CRPs), unclonability (CRP mappings should be physically unclonable), and physical unbreakability (physical PUF modification should result of malfunction or permanent damage to chip as protection of chip integrity) [60]. Advantages of the PUFs are resistant to invasive attacks and have no need for extra programming, testing, and processing power [61].

SRAM cells are resistant to circuit degradation and can be used to build reliable SRAM based PUFs which can be used for authentication and secret key generation [62,63,64]. Huang and Wang [36] have shown that PUF can be utilized for identity verification in order to secure IoT hardware via device authentication. This is achieved by improving a configurable ring oscillator (CRO) PUFs with the latch structure. A unique sub-digital signature of each chip can be generated by performing the challenge-response strategy via CRO-based PUF.

The analysis of PUFs revealed that an exponential improvement will happen soon in the chip area and energy efficiency due to the research efforts [65].

It has been shown in Huth et al. [66] that PUFs can be leveraged by IoT devices to ensure IP protection during software update procedures. Accordingly, an IoT end-device has to prove the erasure of its memory within a time constraint and a PUF binds the newly downloaded software IP to the target platform. Usage of PUFs promises an increase in the security level of the IoT, by enabling low-level security implementations on the things and also by devising cryptography algorithms to perform special tasks (e.g., verification, etc.) [67]. In a similar fashion, fog computing can leverage PUFs that are embedded to the fog gateways and/or IoT end-devices, in order to ensure IP protection during patch updates.

PUF based vulnerabilities in IoT have been studied in many publications [55,68]. Man In The Middle (MITM) attacks are presented in [69], and side-channel attacks in [70,71]. Moreover, improper PUF implementations can result with a backdoor as discussed in [72,73]. All of these attacks mentioned above are crafted for authentication and key generation phases.

### 4.4. Hardware Security Module (HSM)

HSM is a physical computing hardware that safeguards and coordinates digital keys for strong authentication and provides foundations for crypto-processing. These modules are either presented in the form of a plug-in card or an external portable device that can be attached directly to a computer or network server. HSM units can be implemented at FCG’s of the fog computing based IoT network to orchestrate not only the key distribution, but also cryptography related operations such as authentication, encryption/decryption, etc.

**Figure 5:**
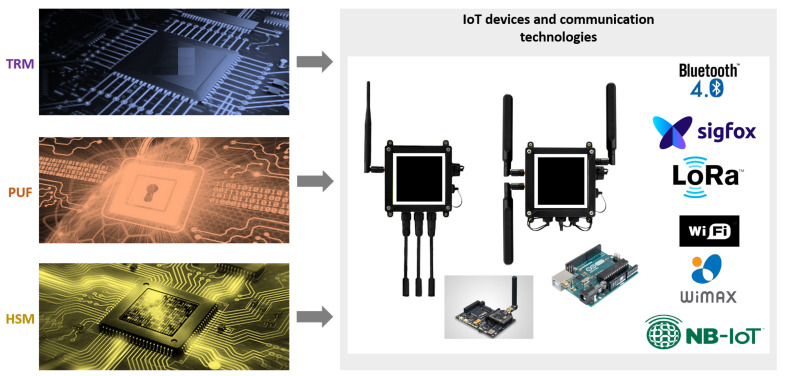
Representation of “Hardware Assisted Security” for fog computing based IoT networks. Logos/images are taken from: Bluetooth [74], Sigfox [75], LoRa [76], Wi-Fi [77], WiMAX [78], NB-IoT [79], Libelium [80], and PI [81].

There are various hardware configurations for IoT end-devices depending on the application they are used for and also the wireless communication technology they are using. For instance, Bluetooth LE, Sigfox, LoRa, WiFi, WiMAX, and NB-IoT are well known wireless communication technologies for the IoT with the radio chip-sets available on the market. Therefore, hardware security solutions will not be generic as they need to consider all the components that are being used in the system. As shown in Figure 5, we project that the above-mentioned hardware security commodity solutions available on the market can be a remedy in providing cyber security for the fog computing enabled IoT networks. TRMs and PUFs are comparably cheaper hardware security solutions and can be used for end-devices of the fog computing enabled IoT networks. However, HSMs are quite expensive devices and to be preferably installed at the FCG’s of the IoT network.

### 4.5. Cost-Analysis of the Hardware Assisted Security

The famous quote by Benjamin Franklin says: “An ounce of prevention is worth a pound of cure.” History has proven this to us many times; if a hardware-based security defense is forgotten and then patched via software, this might not only be costly, but also would cause irreversible performance degradation, or even worse side-effects such as partial or full burn-out of the mentioned hardware if the software fix does not work. As in the case of Intel, a cyber-attack towards a hardware vulnerability (so famed ‘Spectre’ and ‘Meltdown’ vulnerabilities) not only cost Intel millions of dollars (in fixing the old processors via software patches and devising hardware/software prevention mechanism in the newly designed processors) but also a performance degradation up to 15–20% in the software-patched processors that are being used today [82].

In this paper, we do not present hardware-based cyber-security solutions as a replacement-for or competitor to the software-based solutions; on the contrary, we offer hardware assisted security principles along with the software ones. Therefore, they should not be considered as a competing solution, but a complementary one. For instance, storing a password of a software based security solution to a tamper-resistant memory chip. Moreover, one should also consider that hardware-based cyber-security attacks are mostly (maybe completely) thwarted by hardware-based cyber-security solutions not by the software-based ones.

### 4.6. Summary and Recommendations

As mentioned in this section, the PUF-based cyber-defense mechanism does not require intense hardware resources when compared to other solutions such as hardware accelerated encryption, etc. Henceforth, usage of PUF-base based cyber-defense mechanisms is preferably a good option for IoT (FCGs and end-devices) security [68]. TRM chips should be used when trusted hardware needed to ensure that security keys are not tampered with. TRM chips can give strong protection for cloud data centers, mobile-edge, and Fog devices. However, counting on the cost-related benefits, software solutions should be considered for large scale IoT deployments. HSM also can be used as an expensive on-demand defense layer and to be preferably installed at the FCG’s of the IoT network.

## 5. Practical Application Scenarios of Fog Computing in IoT

During the past decade, reduced hardware cost has triggered a rapid increase and an enormous improvement in consumer electronics. Low price microcontrollers and ARM processors are used to design low cost single board computers. ARM is an RISC (reduced instruction set computer) ISA (instruction set architecture) microprocessor widely used in mobile phones and IoT devices. The PIC16(L)F184xx Products (8-bit microcontroller family from Microchip Technology Inc.) price is between $0.53 and $1.04 when ordered bulk [83]. As an example, ARM Microcontroller MCU 32B CORTEX-M3 32KB FL/8KB SRAM is sold on *mouser.com* for $3.20. Owing to the high demand on IoT concept and its applications, single board computers such as Ardunio (Uno Rev3 is starting from $22 on the martket), Raspberry Pi (Pi Zero starts at $5 on the market and Pi 3 Model B+ is available for $35) or Pine64 (A64-LTS is $32.00 on the market) not only are being used by professionals for commercial use, but are also being adopted by hobbyists for any kind of independent studies including elementary-middle-high school projects. Table 4 shows a subset of well known COTS IoT hardware options in a comprehensive manner.

**Figure 6:**
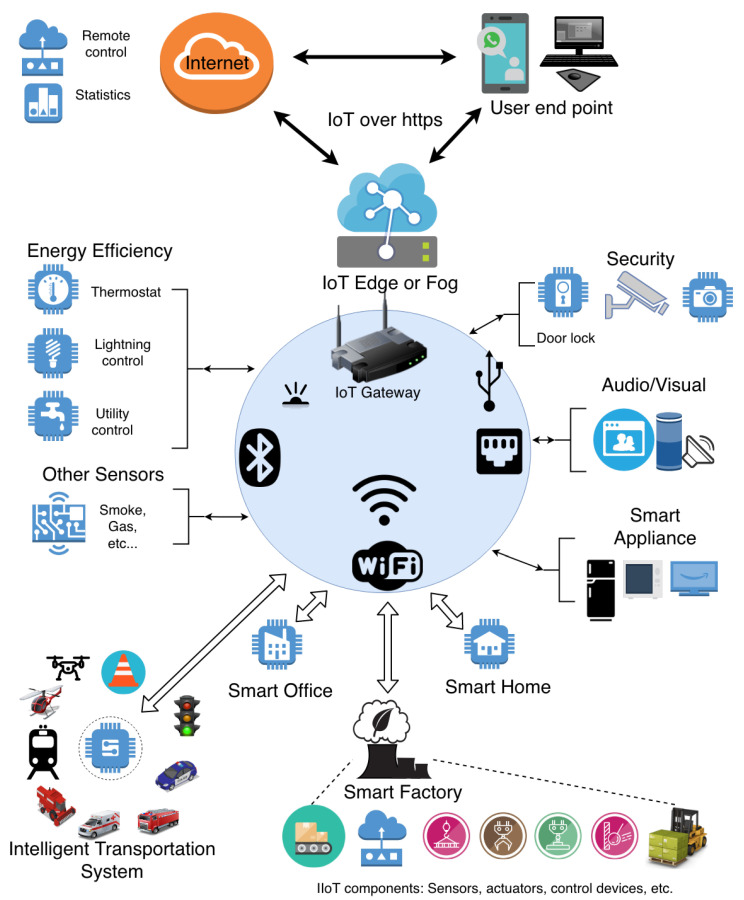
An illustration of four different possible fog computing applications with IoT networks: Smart Office, Smart Factory, Smart Home, and Intelligent Traffic System.

Because of introducing agile response nearby the edge components, we are expecting fast implementation and business growth of fog computing for future IoT applications such as Intelligent Transportation Systems (ITS), smart-factories, smart-cities, etc. Figure 6 presents various possible application fields of fog computing, which are enlisted as follows:

- Smart Homes/Offices: The smart home concept was introduced in 1975 once X10 technology is developed in Scotland [84]. Currently, Zigbee or Z-wave are mostly used for home automation applications. They are basically wireless mesh protocols. The latest Zigbee 3.0 enhances the IEEE 802.15.4 standard by adding security layers. Z-Wave (800–900 MHz frequency) has lower bandwidth than the other standards so it supports a longer distance than the other standards/protocols (up to 100 m). Zigbee has 2.4 GHz frequency and a standard Wifi may have 2.4 GHz or 5 GHz. The higher frequency means a shorter distance for communication. Security-wise, they all support encryption. A Z-Wave network can support a maximum of 232 nodes. (http://www.openzwave.com/dev/) and Z-Wave is limited to 232 devices. Zigbee supports maximum 65,000 nodes connected in a network. Wifi varies based on the router used in a network. To secure smart homes, a security framework utilizing network monitoring and anomaly detection, etc. to address physical, network, and software attacks [85] should be considered.
- Smart Cities: Smart cities can incorporate with the ITS concept through the support of IoT and fog computing to help a sustainable economic development of our world (energy/utility distribution, etc.), safety, transportation (scheduling traffic, signalling systems, etc.), by contributing decision-making with a localization concept. Collecting data from sensors city wide can result in improvement in the efficiency of city services. A redundant task can be found and eliminated (possible financial saving). The end result would be high-quality services at a lower cost. As discussed in Butun et al. [86], smart cities will be vulnerable to many cyber attacks and will need a robust security architecture, for which hardware security assisted fog computing can be employed to help with.
- Smart Factories and Industrial IoT: Automation processes can be improved via data collection with IoT sensors and analyzing these data on the fog environment. Work-flow audit and data collection tasks can be easily accomplished via this methodology. This may result in possible optimization opportunities in IIoT contexts as shown in Forsström et al. [87], in which maintenance prediction or energy efficiency problems can be efficiently solved via the assistance of fog. As also mentioned in Forsström et al. [87], security issues related to IIoT are also on arise and fog-computing based agile cyber-security solutions might be a remedy to this. In addition, to address small and medium sized enterprise needs, a hybrid (hardware-FPGA based middleware security layer, etc., software) security solution should be considered [88].
- Smart Healthcare: IoT and fog can help with improving tools and frameworks in the health industry. Financial improvement, security-surveillance, data collection, and critical medical device-data coordination can benefit. Hardware security assisted fog computing can improve the security of smart healthcare systems. For instance, digital patient records in the hospitals are vulnerable to manipulation attacks. Currently, several vulnerabilities are reported related to the networks and equipment used to transmit and store MRI and CT scan images, which are then sent to radiology workstations through Picture Archiving and Communication System (PACS). The reason of the vulnerabilities is due to the absence of digital signatures and encryption on images when they are stored on the PACS networks. Digital signatures can be attached to the captured images right away at the scanning machines with the help of the fog computing devices mentioned [89].
- Intelligent Transportation Systems (ITS): ITS can be thought as a parent acronym for vehicular networks and vehicular IoT networks. Fog computing is expected to enhance the coverage and decrease the response time of ITS. As mentioned in Munir et al. [10], fog computing can enhance the overall network performance by increasing the response time along with extended coverage. Overall, ITS can benefit from fog computing to increase service quality in the following example scenarios: rapid re-routing of the traffic, fast towing service, emergency services in case of accidents, and, finally, providing necessary evacuation routes in extreme weather events such as hurricanes.

In all of these different scenarios, the common idea is that the end-devices generate a massive amount of data and may need to collaborate with each other to take critical decisions reducing the delay. Hence, an agile response is important and the philosophy of fog computing may help to overcome bandwidth and latency related problems in this manner.

## 6. Conclusions and Future Directions

### 6.1. Lessons Learnt

The proliferation of consumer electronics yielded IoT devices and sensors to approach our close proximity. This is an indispensable development and fog computing will make it further possible due to the inherent advantages it possesses. Throughout this article, we have stressed the implications of using fog computing as a backbone architecture for IoT, especially related to the cyber-security.

Centralized cloud data centers may fail while storing or processing requests from millions of distributed IoT end-devices due to a congested network, high latency in the service, congestion in limited bandwidth, etc. Therefore, as a remedy to this problem, the fog computing concept is projected to be very useful, especially for delay-sensitive applications, such as industrial automation in IIoT. Mobility support, geo-distribution, location awareness, and low latency concepts are important while deploying IoT devices, and fog computing is a strong candidate to help in all of these topics.

This research also has taught us that hardware components of any system, especially fog-computing related IoT systems, are not prone-to/free-from cyber-attacks/hacks/intrusions/ manipulations. Several cyber-attacks against hardware platforms are classified in this work along with defense mechanisms in a summarized manner. As also mentioned by Alioto [65], stronger interaction between chip designers and protocol developers will be indispensable for the next-generation IoT devices to assure a desired level of security at a minimum energy/area cost while at the same time enabling flexibility for future hardware patching.

### 6.2. Future Work

In this article, we discussed integration of fog computing to cloud-based IoT systems. In addition, we elaborated that these IoT systems can leverage commodity hardware such as HSMs and PUFs, or custom tailored SoC hardware, in order to enhance their defense mechanisms against cyber threats, especially in protecting the PII of the users from RE attacks of the ill mannered adversaries.

We foresee that hardware-based cyber attacks will increase in the near future, especially against embedded low-end devices. Thereby, hardware-based cyber-security solutions will be a very useful tool defending against those attacks, more importantly for fog-computing infused IoT networks, especially towards increasing the security abilities of low-end devices which do not have sufficient software/hardware resources to defend themselves against cyber-threats.

## Figures and Tables

**Table 1:** Comparison of cloud and fog and mobile-edge computing concepts [13].

Feature	Cloud	Mobile-Edge	Fog
Access to the network via	Wired (mostly fiber) or wireless	Wireless (mostly cellular)	Wireless (cellular, WiMAX, IEEE802.15, LPWAN, etc.)
Access to the service	Through server	Through BS *	At the FCG *
Agility	Slow	Fast	Fastest
Availability	Mostly available	Mostly available	Mostly volatile
Bandwidth usage	High	Medium	Low
Capacity—Computing	High	Medium	Low
Capacity—Storage	High	Medium	Low
Connectivity	Internet	Many protocols (Figure 3)	Many protocols (Figure 3)
Content distributed to	Edge device	Restricted to BS coverage	Anywhere
Content generator	Man made	Mixed	Sensor made
Content generation at	Central server	BS	FCG
Control	Centralized	Distributed till BSs	Distributed
Data analysis	Long term	Instant/Short term	Instant/Short term
Latency	High	Moderate	Low
Processing/storage at	Center (Server)	Mobile-Edge (BS)	Edge (FCG)
Scalability (Horizontal ^+^)	High	Medium	Low
Scalability (Vertical ^±^)	High	Medium	Low
Security	Weaker	Stronger	Stronger
Mobility	Not supported	Supported	Supported
Number of users	Billions	Millions/Billions	Millions/Billions
Virtual infrastructure at	Enterprise server	Main server	User devices

* BS: Base Station, FCG: Fog Computing Gateway. ^+^ By adding more machine. ^±^ By adding more hardware (CPU, RAM, Storage, etc.).

**Table 2:** Various RF communication technologies for IoT.

Technology	Standard	Frequency	Penetration	Range	Max Data Rate	Channel Bandwidth	Chipset Cost
NFC/RFID	ISO/ICE 18092	13.56 MHz	High	<20 cm	424 kbps	106–424 Mbps	$0.1+
Bluetooth	IEEE 802.15	2.4/2.5 GHz	Low	50–100 m	2 Mbps	2 MHz	$5+
Wi-Fi	IEEE 802.11	2.4/5.0 GHz	Low	100 m	54 Mbps	22 MHz	$1.5-30+
Zigbee	IEEE 802.15.4	868/915 MHz, 2.4 GHz	Low/High	<1 km	250 kbps	2 MHz	$2-20+
DASH7	ISO/IEC 18000-7	433/868/915 MHz	High	0–5 km	167 kbps	up to 1.75 MHz	$3.00+
Weightless	Weightless P/N/W	Multiple	Low/High	5 km	100 kbps	200 Hz–12.5 KHz	∼$2.00
LoRa	Various	868/915 MHz	Low	25 km	50 kbps	125/250/500 kHz	∼$2.00
Ingenu-RPMA	Ingenu-RPMA	2.4 GHz	Low	15 km	20 kbps	1 MHz	rental
SigFox	SigFox	915–928 MHz	Low/High	40 km	100 bps	100 Hz	$0.25+
3G	UMTS/W-CDMA	0.4–3 GHz	Low/High	5–35 km	0.38–21.6 Mbps	3.6–21 Mbps	varies
4G/LTE	3GPP-LTE	0.6–6 GHz	Low/High	5–100 km	100–300 Mbps	100 Mbps+	$6.5+
5G	5GTF/5G-SIG	0.6–4/100 GHz	Low/High	5–150 km	10 Gbps	500 Mbps+	$70+

**Table 3:** Security risk analysis for hardware cyber-threats vs. common security properties for fog-computing based IoT end-devices including attack vector classification. *Legend:* C: Confidentiality, I: Integrity, A: Availability, Auth*: Authentication & Access Control, In: Invasive, NI: Non-Invasive, S: Semi-Invasive.

Threat Category	Severity of the Risk				Vector
	C	I	A	Auth *	
Destroy, remove or steal end-device	None	None	Moderate	None	NI
Device cloning	Moderate	Moderate	Minimal	Significant	In
Firmware replacement	Moderate	Moderate	Minimal	Significant	In
Security parameter extraction by phy. access	Moderate	Minimal	Minimal	Significant	In
Jamming	Minimal	Minimal	Significant	Minimal	NI
Rogue end-device	Moderate	Moderate	Moderate	Significant	In/S
Bit-flipping	Minimal	Moderate	Minimal	Minimal	In

**Table 4:** Comparison of the specs for the subset of the available COTS IoT hardware devices.

	Price	Size	Connectivity	Computation	Power	CPU Specs	Memory	Graphics	Storage
MCU	$0.1–20	Variuos	N/A	Limited	0.29 W	Various	N/A	N/A	N/A
Pi Zero	$10–30	66 × 30.5 × 5	Bluetooth/LAN/Wifi	Low	0.4–1.2 W	*BCM2835 1 GHz	512 MB	Videocore IV	microSD
			1 × microUSB						
			mini-HDMI						
Pi 3	$30–60	85 × 56 × 17	Bluetooth/LAN/Wifi	Medium	1–2 W	1.2 GHz quad-core	1 GB	Videocore IV	microSD
			4 × USB, HDMI			ARM Cortex-A53			
Pine	$15–29	133 × 80 × 19	10/100/1000 Mbps	Medium	1.5–4.1 W	1.2 GHz quad-core	0.5–2 GB	Dual Core Mali	microSD
A64			Ethernet Port,			ARM Cortex-A53		400 MP2	
			2 × USB 2.0			64bit			
Rock64	$25–45	85 × 56 × 18.8	10/100/1000 Mbps	Medium	1.5–4.1 W	RK3328 Quad-Core	1/2/4 GB	ARM Mali	microSD
			1 USB3.0			ARM Cortex A53		450MP2	
			2 USB2.0			64bit		Dual-core	
Jetson	$600	17 × 17 × 5.1	Bluetooth/LAN/Wifi	High	7.5–15 W	Dual-Core Denver 2	8 GB 128-bit	256-core GPU	eMMC 5.1
TX2						64-Bit	LPDDR4	NVIDIA Pascal	
Libelium	$130	73.5 × 51 × 13	Mini USB	Low	< 15 W	ATmega1281	N/A	N/A	SD Card
Arduino	$11–77	Various	Micro/Mini/Regular	Low	≥ 0.29 W	8MHz to 400Mhz	KB < 64 MB	N/A	EEPROM
			USB						
TelosB	$99	81.9 × 32.5 × 6.55	USB	Low	≥ 0.075 W	8 MHz TI MSP430	KB < 1 MB	10 KB	Ext Flash

## Abbreviations

The following abbreviations are used in this manuscript:

BS	Base Station
CBS	Cloud-Based Services
CIA	Confidentiality, Integrity, Availability
CoT	Cloud of Things
CRA	Code Reuse Attack
CRO	Configurable Ring Oscillator
CRP	Challenge Response Pair
DoS	Denial of Service
DDoS	Distributed Denial of Service
FaaS	Fog as a Service
FCG	Fog Computing Gateway
HSM	Hardware Security Module
IaaS	Infrastructure as a Service
IDS	Intrusion Detection System
IoT	Internet of Things
IIoT	Industrial Internet of Things
ISA	Instruction Set Architecture
ITS	Intelligent Transportation Systems
LBS	Location-Based Services
MEC	Mobile Edge Computing
PaaS	Platform as a Service
PACS	Picture Archiving and Communication System
PII	Personally Identifiable Information
PUF	Physically Unclonable Functions
QoS	Quality of Service
RE	Reverse Engineering
RISC	Reduced Instruction Set Computer
SaaS	Software as a Service
SoC	System on a Chip
TaaS	Things as a Service
TRM	Tamper Resistant Memory
VM	Virtual Machine
